# Listening to earthworms burrowing and roots growing - acoustic signatures of soil biological activity

**DOI:** 10.1038/s41598-018-28582-9

**Published:** 2018-07-06

**Authors:** Marine Lacoste, Siul Ruiz, Dani Or

**Affiliations:** 1 URSOLS, INRA, 45075 Orléans, France; 2 Soil and Terrestrial Environmental Physics, Institute of Biogeochemistry and Pollutant Dynamics, ETH Zurich, Zurich, Switzerland

## Abstract

We report observations of acoustic emissions (AE) from growing plant roots and burrowing earthworms in soil, as a noninvasive method for monitoring biophysical processes that modify soil structure. AE emanating from earthworm and plants root activity were linked with time-lapse imaging in glass cells. Acoustic waveguides where installed in soil columns to monitor root growth in real time (mimicking field application). The cumulative AE events were in correlation with earthworm burrow lengths and with root growth. The number of AE events recorded from the soil columns with growing maize roots were several orders of magnitude larger than AE emanating from bare soil under similar conditions. The results suggest that AE monitoring may offer a window into largely unobservable dynamics of soil biomechanical processes such as root growth or patterns of earthworm activity - both important soil structure forming processes.

## Introduction

Soil is a critical living system that supports key biogeochemical cycles, a rich array of ecological processes, and contributes to numerous ecosystems services^1–3^. The complex aggregation and arrangement of mineral and organic soil constituents give rise to an important and fragile trait called soil structure^4^, considered central to soil agro-ecological functioning^5,6^. Soil structure results from a dynamic equilibrium^5^ that may take decades to build but seconds to alter (e.g., passage of a heavy vehicle), and reported recovery times from such damage range from month to centuries^7^. The maintenance of favorable soil structure for agricultural production is particularly challenging due to its sensitivity to tillage and other aspects of crop management. For example, it is estimated that about 68 Mha of land worldwide are affected by soil compaction^7^, highlighting the importance of soil structure management for sustainable agricultural production and environment protection^2^.

For both natural and managed soils, biological activity is important for generating and sustaining favorable soil structure^6^. The resulting soil structure reflects intricate feedback processes between soil biota and their environment; it comes as no surprise that biological activity is considered as one of the five central soil forming factors^8^ and as a primary factor of soil structure formation^9–11^. The soil-biological interactions span all scales, from the micron scale where structural heterogeneity facilitates coexistence of potentially competitive microbial consortia and thus supports large biodiversity of microbial life^6,12,13^, to pore scale (mm to cm) where the root penetration can enhance preferential flows by the creation of new pores and then impact the soil water regime^9^. Plant roots play a central role in forming suitable conditions for life in soil^9,14^, not only by improving aeration and infiltration but also by creating conditions favorable for formation of biological hotspots^15^. Another important biological agent for soil structure formation are earthworms, often referred to as “ecosystem engineers”^10^. Earthworms form burrows through the soil seeking carbon rich zones^14^ (e.g. dead plant residues), and these burrows serve as preferential paths for water flow and gas transport^16,17^. The network of biopores facilitates plant root growth by providing direct access to oxygen and less resistant mechanical paths^18^.

Available observation methods for soil structure quantification often overlook these highly dynamic biophysical processes. A few methods such as rhizotron imaging provide certain insights into changes in root-soil interactions^19–22^, yet the method is qualitative and limited to prescribed window of observation and thus is of limited value for inferences of root system dynamics^23^. Modern application of X-ray computed tomography provide insights into describing soil structure^24,25^ and consequences of soil bioturbation by earthworms^26^ and plant roots^18,27^. However, such methods remain lab bench based and are not yet available for monitoring such dynamic processes *in situ*.

Alternatively, monitoring acoustic emissions (AE) from soil provides dynamic and *in situ* information with potential to circumvent shortcomings of other conventional measuring techniques. The utility of passive AE measurements have already been demonstrated for applications ranging from structural engineering^28^ to geoscience^29–32^. In soils, AE are generated by a sudden release of elastic energy due to modification of grain contacts or sudden soil aggregate rearrangement, friction between aggregates and grains, changes in interfaces between gas and liquid surfaces, and crack formation^33^. The resulting elastic waves are characterized by high frequencies (1–100 kHz) beyond the audible range. However, associated AE can be amplified and monitored using highly sensitive piezoelectric sensors that register acoustic events exceeding an amplitude threshold and various other metrics of the process.

We hypothesized that AE resulting from growing plant roots and burrowing earthworms in soil could be measured *in situ*. Controlled experiments were conducted in which we monitored AE produced by earthworm activity and maize roots growing in glass cells filled with soil. We then monitored plant roots growing into soil columns equipped with glass acoustic waveguides to explore the feasibility of AE monitoring for field applications.

## Results

### Acoustic emissions from burrowing earthworm in soil

**Figure 1:**
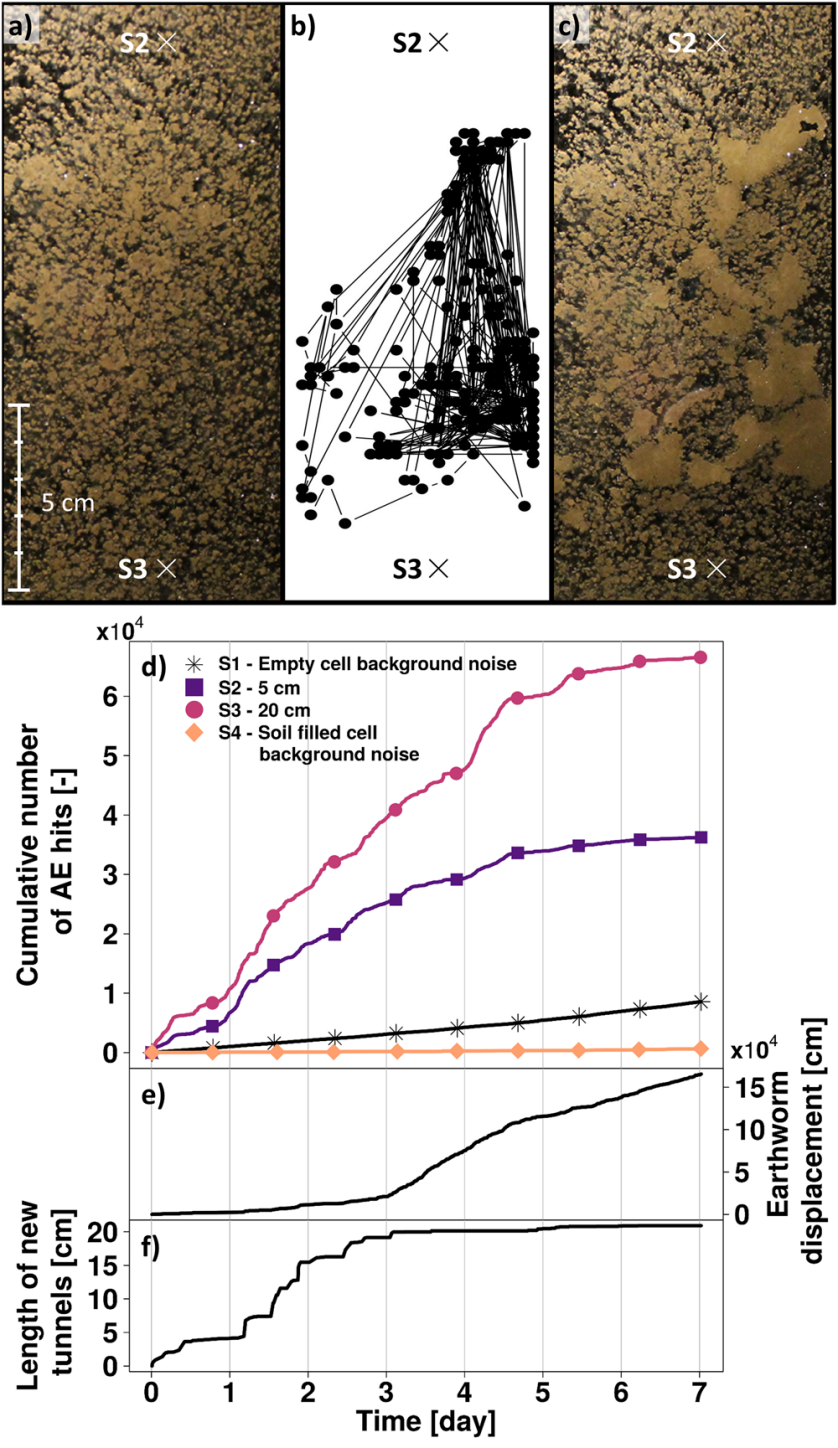
AE monitoring and earthworm activity in a soil filled glass cell. Time-lapse images were taken from the front face of the glass cell for the full duration of the experiment (**a**) beginning and–(**c**) end of the experiment), where X’s indicate the locations of the acoustic sensors. The initial packing (**a**) was augmented by movement of the earthworm (trajectories illustrated in **b**) culminating in a final perturbed soil state. (**c**) Cumulative acoustic events were monitored (**d**) during the seven days experiment. The results for sensors S2 to S4 are given after background noise filtering from sensor S1. Total cumulative earthworm motion (**e**) and total length of new tunnels (**f**) were determined based on the activity monitored in (**b**).

AE were generated over seven days by an individual earthworm in a glass cell (8 mm gap) filled with freshly packed silt loam soil (Fig. 1). The cell design facilitated direct imaging of the earthworm activity and the burrows formed during this period from initial soil state (Fig. 1a) to the final soil perturbed state (Fig. 1c). AE were measured on three glass cells, one containing one earthworm and two control glass cells (one empty and one filled with soil but still without an earthworm, Fig. 1d). Additional details are provided in the *Materials and Methods* section. The recorded AE events (termed “hits”) in the earthworm cell were about an order of magnitude higher than AE recorded in the control cells, suggesting that AE were dominantly generated by earthworm burrowing activity. The AE events recorded in the earthworm cell by the upper and lower sensors (S2 and S3) increased in magnitude during the first four days and subsequently plateaued. In contrast, the cumulative movement of the earthworm based on visual images (Fig. 1e) gradually increased during the first three days, and then rapidly rose afterwards. The correlations between earthworm motion and AE were relatively low with R^2^ of 0.24 and 0.09 for sensors S2 and S3. However, tunnel construction (Fig. 1f) and AE showed a better trend agreement, resulting in a higher correlation between daily tunnel creation and daily cumulative AE events with R^2^ of 0.90 and 0.62 for AE sensors S2 and S3 (See Supplementary Information online for detail). The visually observable earthworm activity was nearly uniformly distributed between the top and bottom of the glass cell, with a slight bias towards the bottom part of the cell (closer to sensor S3). This resulted in similar acoustic signatures measured by both sensors, and slightly more AE events recorded by sensor S3. This experiment was re-run to examine repeatability of results (Supplementary Fig. S4), where the earthworm activity was localized near the lower sensor S3 (Supplementary Fig. S4b,c). The resulting AE events were captured by the sensor near the location of the burrowing activity (sensor S3), supporting the hypothesis regarding a correlation between measured AE signals and earthworm burrowing activity. The replicate results are detailed in the Supplementary Information.

### Acoustic emissions from plant root growth in a glass cell

**Figure 2:**
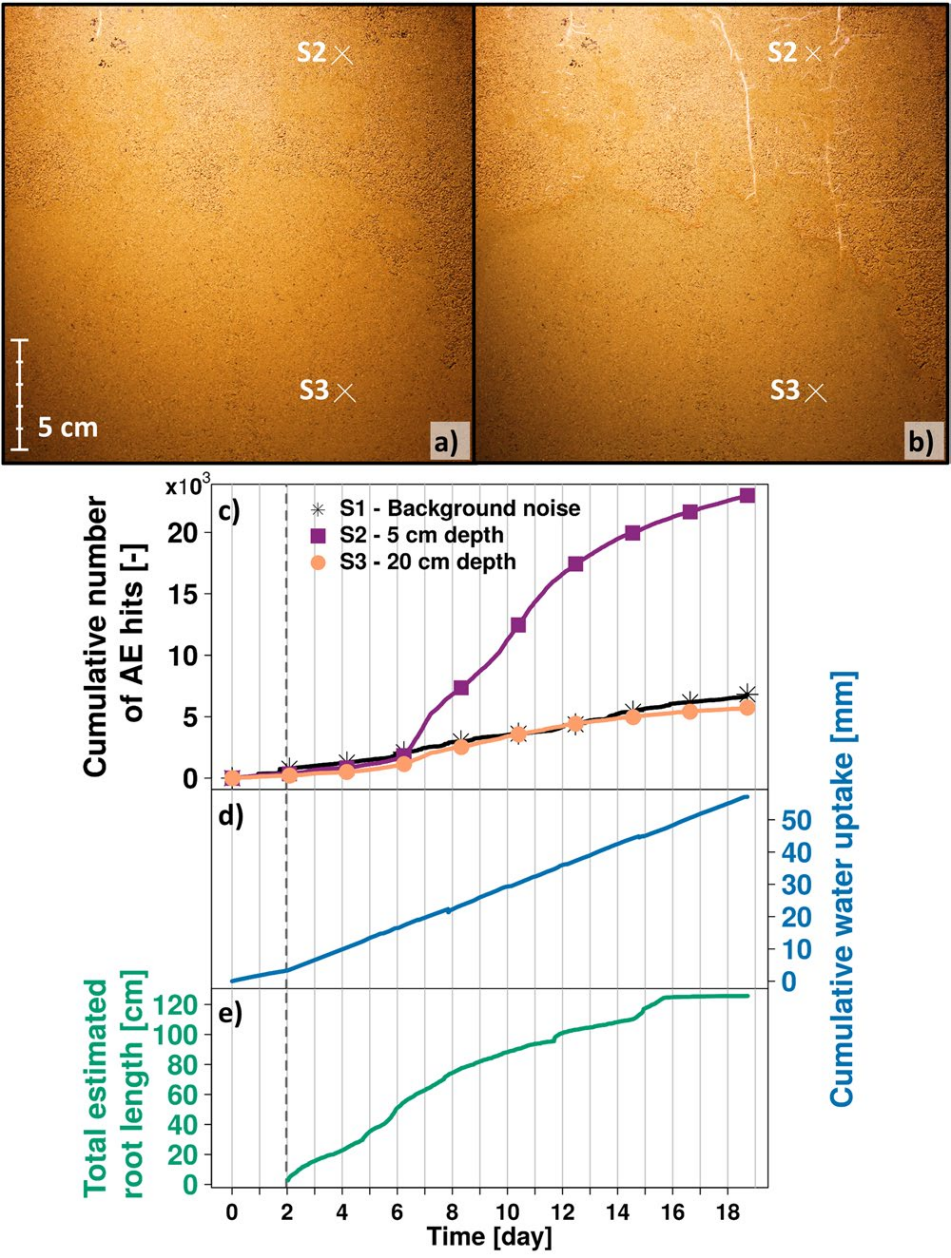
AE monitoring during maize roots growing in a soil filled glass cell. Time-lapse images were taken to monitor maize roots growing in the glass cell from the day the geminated seeds are planted (**a**) to the last day of the experiment (**b**) where X’s indicate the locations of the acoustic sensors. Cumulative number of acoustic events were monitored for the three separate acoustic sensors. (**c**) The results for sensors S2 and S3 are given after background noise filtering from sensor S1. Simultaneously, the cumulative water uptake was also monitored (**d**) as well as the estimated total root length (**e**) determined with time-lapse images (**a**,**b**). The vertical dashed line (**c**–**e**) denote the time when germinated seeds were planted in the glass cells (two days after the beginning of the experiment).

AE generated during root growth in a glass cell (12 mm thick) filled with sandy soil were monitored over 19 days (Fig. 2). Three germinated maize seeds (*Zea mays*) were planted near the surface of the glass cells on day 2 and the root growth was visually monitored for the following 17 days (Fig. 2a,b). The AE events generated over the period were measured at 5 and 20 cm depth (sensors S2 and S3), and a control sensor (S1) was used to record background AEs (Fig. 2c). Additional details are provided in the *Materials and Methods* section. The cumulative AE events remained relatively low for all three sensors for the first five days. On the sixth day, the rate of AE events increased for sensors S2 and S3, with a particularly steep increase for the upper sensor S2. After day 15, the AE events measured by sensors S2 and S3 slowed and began to plateau. The sensor located closer to the soil surface (sensor S2), the area of the glass cell where more root growth was observed, recorded significantly higher number of AE events. Soil evaporation rates before seeds introduction was 1.7 mm day^−1^, and evapotranspiration rates rose to nearly constant value of 2.9 mm day^−1^ for the subsequent 14 days (Fig. 2d). The trends of the cumulative water uptake do not follow the trends in AE events. Time lapse images have shown rapid and numerous root growth between day 2 and 10 (Fig. 2e). The total root growth slowed down and plateaued around day 15 with an observable total root length of about 126 cm. The plateau in root growth corresponded well with the slowing down of recorded AE events (see Supplementary Information online for detail). This experiment was re-run to check for repeatability of results. The replicate experiment (Supplementary Fig. S5) showed similar trends involving filtered acoustic signatures (the difference between the measured AE and the background noise). The cumulative measured AE signals rise to a plateau (Supplementary Fig. S5a) similar to the estimated root length (Supplementary Fig. S5c). The replicate results are detailed in the Supplementary Information.

### Acoustic emissions from growing plant roots using waveguides in soil columns

**Figure 3:**
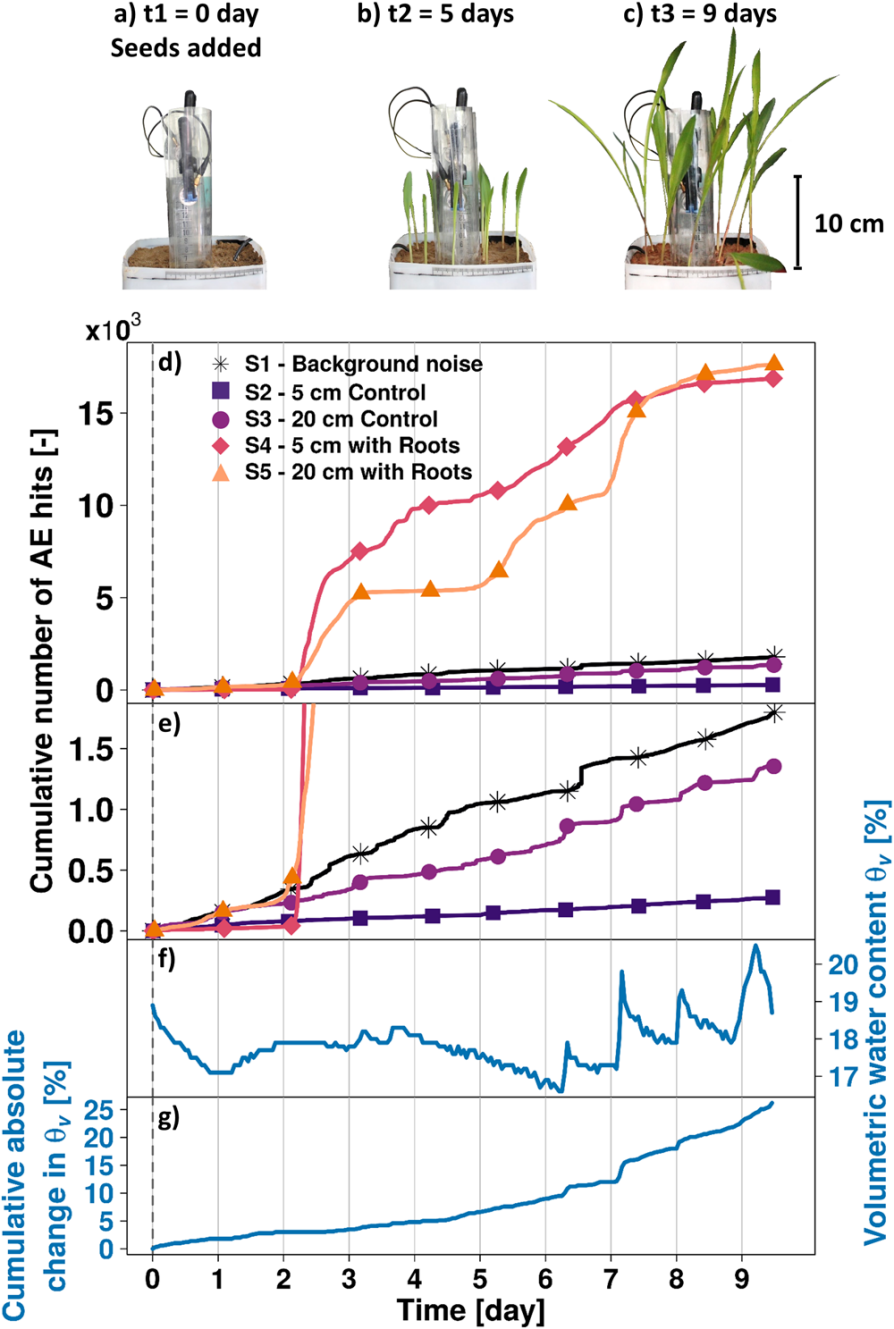
Monitoring AE generated by maize roots growing in a soil column. Pictures of the maize stem growth during the experiment were taken daily. (**a**–**c**) Cumulative number of AE hits (events) were recorded in the control bare soil column (sensors S1 and S2) and in the column with maize (sensors S4 and S5) (**d**). The results for sensors S2 to S5 are given after background noise filtering from sensor S1. The image was magnified (**e**) in order to more clearly see the trends of S2 and S3 on the controlled column. Volumetric water content (θv) in the column without maize was measured (**f**) and the cumulative absolute change in θv over time was computed (hourly time-lapse) (**g**) representing the absolute change in water content in the column, independently of drying or wetting processes. The vertical dashed line (**d**–**g**) denote the time when germinated seeds were planted in the glass cells (at the beginning of the experiment).

AE events resulting from maize roots (*Zea mays*) growing into a squared based soil column (base: 15 cm × 15 cm; height: 20 cm) were monitored. Twelve germinated seeds were planted at the top of the soil column and their growth was monitored for nearly 10 days (Fig. 3). Given the challenge of directly observing root lengths in the opaque soil column, we obtained daily measurements of plant stem heights as a surrogate for root growth. Stem heights reached 10 cm five days after planting and about 25 cm after nine days (Fig. 3a–c). AE events were monitored in two soil columns, one containing the growing maize plants, and an identical control column with only soil (see the *Materials and Methods* section for detail). At the end of the experiment, the cumulative number of AE events registered in the column with maize was several orders of magnitude larger than the AE from the bare soil column under similar conditions (17 × 10^5^ events from the soil with plants and 10^3^ from bare soil; Fig. 3d).

During the first two days of the experiment, similar AE event rates were registered for sensors S2 and S4 (5 cm depth for bare soil and the maize column), and for sensors S3 and S5 (20 cm depth) (see Supplementary Table S3 in Supplementary Information). Subsequently, a peak of AE events were observed in the column with maize plants (sensors S4 and S5) while AE rates in the control soil column remained constant. The AE rates fluctuated in the column with maize, alternating period of higher activity with period with low AE, ultimately leveling off after the seventh day of the experiment. While the control sensors S2 and S3 recorded events in a manner that correlated with the water movement in the system (Fig. 3e–g), there were no obvious correlations with water movement in the column with maize roots. The first spike in AE events for sensors S4 and S5 occurred when the soil water content was constant. The AE event peaks observed in the control bare soil column (Fig. 3d) were considerably lower than observed in the column with maize plants (see Supplementary Information online for detail). This experiment was re-run to check for repeatability of results. While our replicate experiment (Supplementary Fig. S7) generated fewer AE events, the detected signatures were of the same order of magnitude and clearly higher than the AE generated in the bare soil column. They were also uncorrelated with water movement, suggesting that the source of AE signals was root growth. The replicate results are detailed in the Supplementary Information.

## Discussion

The study aimed to explore the potential of passive AE monitoring of soil biophysical processes associated with root growth and earthworm burrowing that contribute to soil structure generation. The results support the hypothesis that these soil bioturbation processes generate distinctive and measurable AE events that were highly correlated with observed activity. For each experiment, the AE produced by background processes in control setup (cells or columns) without the biological agents were also monitored. The resulting AE patterns from these controls were clearly different from cells and column containing biological agents.

For the earthworm experiments in the glass cells, water was not resupplied and the surfaces were sealed to reduce evaporation. We thus attribute AE events primarily to earthworm activity such as burrowing, movement in existing tunnels and possibly soil ingestion. The results have shown that the daily AE rate was strongly linked with creation of new tunnels, and less correlated with earthworm movement in soil (Fig. 1), likely due to the re-use of pre-existing tunnels.

Similar correlations between plant root growth and daily AE rate were observed during the plant glass cell experiment (Fig. 2). The temporal trends in evaporation did not correspond to AE events recorded in the same cell, whereas root growth trends were strongly correlated with AE event rate. AE events were delayed (in time) relative to observed root extension probably due to sensor positioning on the cell wall opposite to the observation wall. In other words, root growth was visually monitored at the front of the glass cell, whereas AE sensors were placed on the back.

The results from the soil column experiment (Fig. 3) yielded similar AE dynamics as those obtained in the glass cell, with a high AE event peak occurring two days after the germinated seeds were planted. The number of AE events recorded from the soil column with growing maize roots were several orders of magnitude higher than the AE in the bare soil column, suggesting that the generation of AE was dominated by the root-soil-water mechanical interactions rather than the movement of water alone. The results obtained in the replicate experiments corroborated these observations, as the recorded AE signals were correlated to the respective biological activities (earthworm burrowing and root growth). The AE signals in the replicate experiments exhibited similar trends but the values were not identical to those recorded in the first run experiments. We note that the observed biological activities were also different, in particular: the length of new tunnels created and the displacement rates and locations for the earthworm experiment, the lengths and roots density for the plant root experiment, and the number of growing plants for the plant root column experiment. The observed differences in AE signals were consistent with differences in the biological activity, illustrating the potential of the AE method to monitor and potentially quantify different rates of biological activity in soil and distinguish various processes linked to soil structure formation.

The proposed acoustic monitoring method in soil holds a promise for *in situ* non-invasive scrutiny of soil structure alteration (formation of biopores and/or destruction of soil aggregates) by bioturbation by plant roots, earthworm, and other fauna. Passive acoustic monitoring is a dynamic observation method that may provide new insights into important processes that are not easy to observe (root growth, earthworm activity, etc.), and thus help identify conditions that promote or suppress them. Apart from soil structure dynamics, this method could also help in creating knew knowledge in plant and earthworm ecology. We note that the aim of the study was not to faithfully reproduce *in situ* earthworm behavior and plant root development (with proper thermal gradients, no light exposure etc.), but to experimentally establish whether AE signals could be measured and allow monitoring their activities in relation to soil structure evolution. Therefore, aspects of the experiments may have influenced the behavior of these biological agents and limit generalization of ecologically-related conclusions. We are especially concerned that the permanent illumination of the glass cells containing the earthworms may have affected their behavior (displacement speed, burrowing activity, etc.). However, with an appropriate setup or through *in situ* measurements, passive AE could then be used to capture the dynamics of root growth, and help answer question to improve cropping systems in the context of precision agriculture. For example, it could allow the determination of when plant roots are growing, when there is water movement through the soil matrix, etc. Considering earthworm ecology, passive AE could contribute to specify where and when they are active, and to make a distinction between the creation of new tunnels (soil structure modification) and displacement in existing tunnels. As the earthworms are very active in the first few centimeters of soils, in the rhizosphere, the method could also be applied to study the interaction between roots and earthworms.

While the replicate experiments show promise that this method may have utility for broader applications, the results of this study are still exploratory and the method requires further development and refinement. The sensitivity of the method to soil texture, soil water content, plant species, etc. should be investigated. For example, the soils used in this study were selected for their potential to produce AE events (silt loam soil for the earthworm experiment and sandy soils for the plant roots experiments). We expect that AE monitoring would be less effective in very wet and fine textured soils (i.e. wet clayey soils) as confirmed in a preliminary penetrometer tests associated with AE monitoring (not reported). It is possible, however, that even when bioturbation activity occurring under wet conditions is not observable using passive AE in clayey soils, other soil structure processes such as crack formation during wetting/drying cycles would be captured by such method. An important aspect related to the use of acoustic waveguides is the high attenuation of AE events in soil. We envision using specially designed acoustic waveguides within plant root zones to extend the range of detectability^33^. Additionally, new methods for AE detection are becoming available and the adaptation of fiber optical sensors^34^ for soil biophysical monitoring appears entirely feasible. In conclusion, additional studies are required to address the following aspects: (1) distinction between different types of AE generation processes, (2) the range of conditions favorable for AE monitoring, (3) application of the AE method in field experiments, and (4) the development of affordable, portable and robust AE systems and sensors for routine field applications.

## Materials and Methods

### Brief overview of acoustic emission generation during soil bioturbation

Generally, acoustic emissions originate from a sudden release of elastic energy stored in grain contacts, force chains, or air-water interfaces. The energy travels as an elastic wave and is registered by piezo-electric (or fiber optical) sensors. The travel distances of elastic release events depend on soil mechanical properties and saturation degree, both contribute to signal attenuation where typical AE rarely travel beyond a fraction of a meter^35^.

Soil bioturbation by earthworms and growing plant roots alter the arrangement of grains, particularly collision between large (sand) grains, grains friction and crack formation (primarily by expanding plant roots) that produce AE events – similar mechanisms were discussed at length in a recent review^33^. The measurement of these events is critically dependent on capturing these high-frequency signals (10–100 kHz) before they attenuate, hence, the use of rigid acoustic waveguides (metallic or glass) as used in this study that transmit local AE to large distances (as in the case of growing roots within a soil volume).

It is important to distinguish earthworms from roots in terms of potential for AE generation. Earthworms burrow at high rates (100–500 *μ*m s^−1^) relative to extending plant roots (0.1–0.2 *μ*m s^−1^)^36^. We thus expect earthworms to impart higher rates of mechanical energy into soil deformation and thus accentuate release of elastic energy events. In contrast, plant roots will impart mechanical energy at lower rates (slower growth rates), however, the large number of root tips per soil volume may compensate for the low local rate in terms of probability of AE release events. To enhance the potential for AE signatures, we selected a coarse growth medium (sandy soil) and used acoustically conductive wave-guides to better detect the cumulative AE generated by a growing root system. Finally, we note that plant roots may exerting large radial pressures^37^ (>1000 kPa) in relatively dry soils that may induce cracks and contribute to detectable AE’s.

### Acoustic acquisition sensors and monitoring system

**Figure 4:**
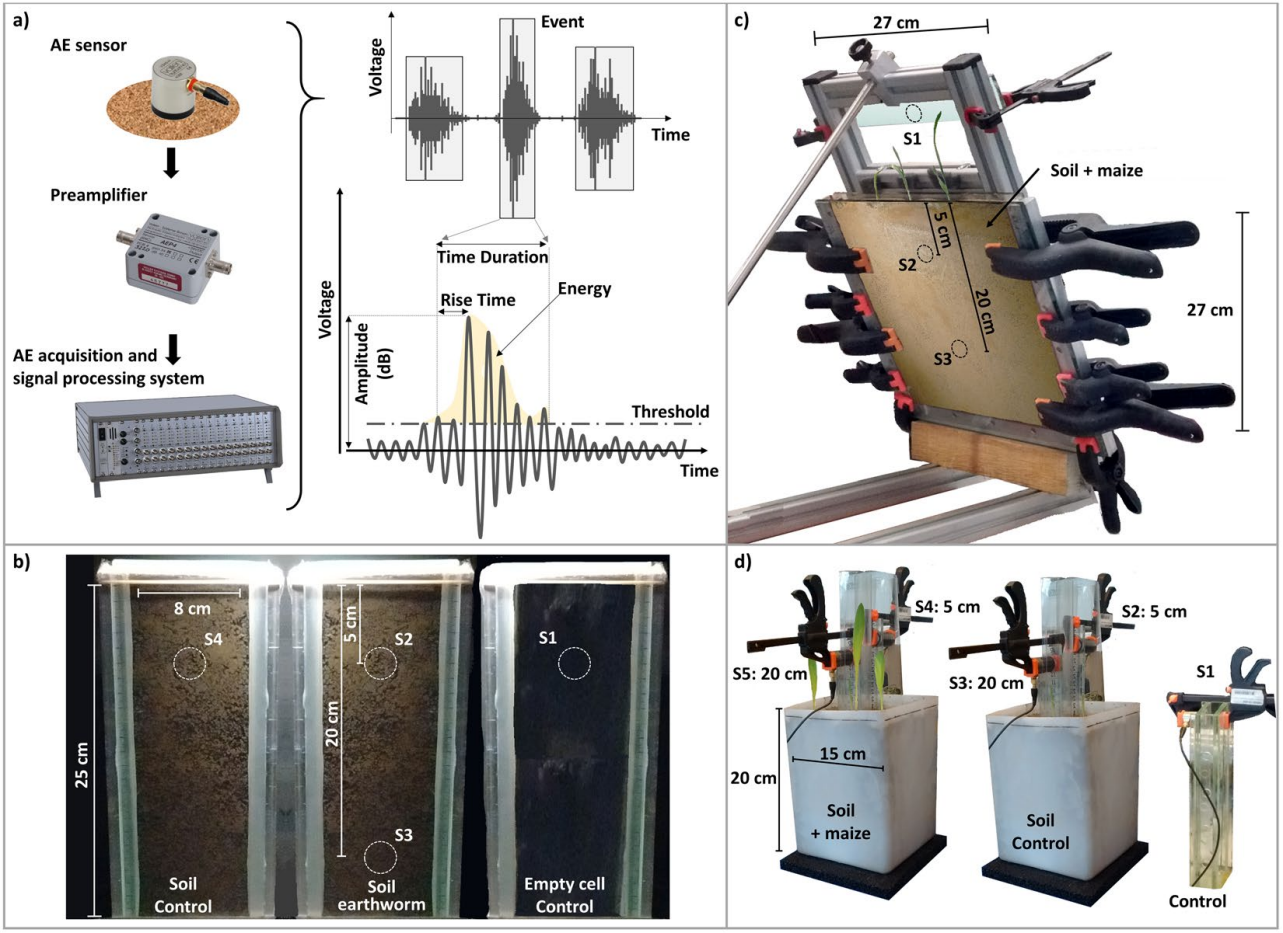
Experimental equipment and assembly. (**a**) AE sensors, preamplifier, and acquisition system used for monitoring acoustic emissions. The acoustic events were defined as acoustic signals that fluctuate over a prescribed threshold for an extended time duration. (**b**) AE monitoring of earthworm activity was conducted by mounting the AE sensors to the backs of a soil filled glass cell. One experimental glass cell contained soil and one earthworm, and two controls were used (one in an empty glass cell and another in a soil filled cell). (**c**) AE monitoring of maize root growth in glass cell with two sensors mounted on the back end of the cell (one at 5 cm depth and another at 20 cm depth) with a control on a fixed piece of glass on the external frame. (**d**) AE monitoring maize root growth in soil column. Sensors Sx mentioned on Figs 1–3 are localized on each setup.

A Vallen AE monitoring system (http://www.vallen.de/) was used for the three experiments reported here. The system comprise of: (i) a AMSY-5 digital multi-channels AE-measurement system, allowing parallel AE recording with several sensors, life time monitoring of the AE recording, data filtering and processing, (ii) VS45-H sensors, which are passive piezoelectric AE-sensors with a wide frequency response, characterized by a peak at 280 kHz. They can be used in the frequency range from 40 kHz to 450 kHz, and (iii) wide-band AEP4 preamplifiers, with a gain of 34 dB (Fig. 4a).

In the glass cell experiments, the AE sensors were in contact with the background glass face. For the soil column experiment, glass acoustic waveguides were used to ensure a good contact between the soil and the AE sensors and limit the attenuation of acoustic waves in soil. The contact between the AE sensors and the waveguides were obtained using a silicone acoustic couplant. A minimal amplitude threshold of 30.6 dB was applied. AE events were analyzed in term of number of events in time (cumulative AE events in time, AE hourly rates), which reflect the temporal dynamics we were interested in. However, the AE acquisition system also described the AE events by recording parameters such as peak amplitude, energy, rise time, duration and counts.

### Experimental design for acoustic measurements of earthworm activities in a glass cell

AE monitoring of earthworm activities was done using three glass cells (inner space: 25 cm height, 8 cm wide, 0.8 cm thick; Fig. 4b). One glass cell was kept empty to monitor the background noise occurring during the experiment without the influence of either the soil or earthworm activity (sensor S1). Two glass cells were filled by a silt loam soil (Uetliberg), sieved at 2 mm, with a bulk density of 1.1 g.cm^−3^ and an initial volumetric water content (VWC) of 38%. These cells were closed to minimize soil water evaporation and there was no water input in the cells during the experiment. One soil filled glass cell was used to monitor earthworm activity with two AE sensors maintained in contact with the glass cell at 5 and 20 cm from the top soil surface (sensors S2 and S3); the other cell was used as a control (i.e. soil without earthworm; sensor S4).

An endogeic earthworm species, *Octolasiun cyaneum*, was used. The earthworm was kept in the Uetliberg soil for acclimation prior to the experiment. Then it was placed on a filter paper for a two days fasting period before its introduction into the experimental cell. At the beginning of the experiment, one earthworm was introduced in a small PVC tube that was fixed on the side of the glass cell with direct passage to the soil. The three glass cells were placed into a cold chamber where the temperature was kept around 13 °C. LED lights were used to continuously illuminate the setup during the experiment and allow visual monitoring of the earthworm motion by the use of a Canon EOS 1100D camera (Canon Inc., Tokyo, Japan; an image was taken every 10 min). Only one earthworm was introduced in the glass cell at a time. Running the experiment with several earthworms would have made it difficult to distinguish between the trajectories of an individual earthworm.

### Experimental design for acoustic measurements of plants root growth in a glass cell

AE monitoring of root growth was initially conducted in a glass cell. The glass cell (inner space: 27 cm height, 27 cm wide, 1.2 cm thick; Fig. 4c) was filled with a sandy soil (Winzlerboden), sieved at 2 mm, with a bulk density of 1.4 g.cm^−3^. The Winzlerboden soil was chosen because of its high sand content, to favor the production of AE during the root growth. The glass cell was linked to a hanging water bottle to ensure a constant water content (VWC around 30%). Three maize seeds, germinated prior to the experiment, were planted at the soil surface two days after the beginning of the experiment. The glass cell was fixed on a tilted frame, exploiting root gravitropism in order to better visualize root growth. To monitor the background noise occurring during the experiment, one AE sensor was fixed on a glass plate maintained on the same frame as the glass cell, but not in contact with the soil (sensor S1). Two AE sensors were placed in contact to the back side of the glass cell filled by soil, at a distance of 5 and 20 cm from the top soil surface (sensors S2 and S3). A LED light was used to illuminate the setup and allowed visual monitoring of the root growth by the use of a Canon EOS 1100D camera (Canon Inc., Tokyo, Japan; an image was taken every 30 min).

### Experimental design for acoustic measurements of plants root growth in a soil column

AE monitoring of root growth was then conducted in a square based soil columns (inner space: 20 cm height, 15 cm wide, 15 cm thick; Fig. 4d). Two columns were filled with the sandy soil (Winzlerboden), sieved at 2 mm, with a bulk density of 1.2 g.cm^−3^ and a VWC of 18% (i.e. close to the water content at water field capacity at pF 2). One column was used for root growth monitoring, and the other as a control (bare soil). An AE sensor was placed on an empty planar cell near the two columns in order to monitor background noise (sensor S1). Two AE sensors were used for each soil column, fixed on waveguides made from glass plates (0.6 cm thick, 3 cm wide and 15 or 30 cm long). These waveguides were inserted in the soil columns at a depth of 5 and 20 cm (sensors S2 and S3 for the control column, sensors S4 and S5 for the column with maize plants). To ensure a sufficient maize growth, twelve maize seeds were added in one column at the beginning of the experiment (after two days of germination). In order to supply water to the plant roots and minimize the acoustic interference of water movement, a TDR soil moisture sensor (EC-5, Decagon Devices, http://www.decagon.com) was imbedded in the control column and was coupled to an irrigation system where water supply (from the bottom of the columns) was controlled by an electronic valve to insure the column was maintained at a volumetric water content of 18%. The same volume of water that was used to irrigate the control column was also supplied to the column with the plant roots. It was not possible with this setup to follow the root growth, but the leaf growth was measured daily and monitored by the use of a Canon EOS 1100D camera (Canon Inc., Tokyo, Japan; an image was taken every hour).

### Data availability

The datasets generated and analyzed during the current study are available from the corresponding author on reasonable request.

## Electronic supplementary material

Supplementary Information

Supplementary Video S1

Supplementary Video S2

Supplementary Video S3
