# Supplementary material for: Listening to earthworms burrowing and roots growing - acoustic signatures of soil biological activity
Source: Sci Rep. 2018 Jul 6;8:10236. doi: 10.1038/s41598-018-28582-9 (PMC6035217; doi:10.1038/s41598-018-28582-9)
Supplement: Supplementary file 1 — Supplementary Information [file 41598_2018_28582_MOESM1_ESM.pdf]

Listening to earthworms burrowing and roots growing - acoustic signatures of soil biological activity

Marine Lacoste[a*], Siul Ruiz[b], Dani Or[b]

[a] URSOLS, INRA, 45075 Orléans, France

[b] Soil and Terrestrial Environmental Physics, Institute of Biogeochemistry and Pollutant Dynamics, ETH Zurich, Zurich, Switzerland

***** Corresponding Author: Marine Lacoste, URSOLS, INRA - Centre de Recherche Val de Loire, site d'Orléans, 2163 avenue de la Pomme de Pin - CS 40001 Ardon, 45 075 Orléans Cedex 02, France, +33 2 38 41 80 32, marine.lacoste@inra.fr

Supplementary Information

All data analysis for the three experiments were carried out in R[1], which include acoustic emission data and data extracted from image monitoring.

Soil structure dynamics and earthworm activity

The acoustic emission (AE) rates were higher in the glass cell with earthworm than in the two control glass cells (Supplementary Table S1). In the two control cells, the AE rates stayed constant during the experiment (mean AE of 1062 and 68 AE.day^{-1} for sensors S1 and S4 during the first four days; mean AE of 1429 and 131 AE.day^{-1} for sensors S1 and S4 during the last three days). The soil filled control cell (sensor S4) measured few events. The AE rate was higher in the glass cell with earthworm during the first four days (mean AE of 7346 and 11940 AE.day^{-1} for sensors S2 and S3) than during the last three (mean

AE of 2279 and 6259 AE.day^{-1} for sensors S2 and S3). To accompany the acoustic measurements, earthworm burrowing activity was monitored using time-lapse imaging. Despite the narrow spacing of the cell (0.8 cm of thickness), it was difficult to image the earthworm when it was in contact with the back of the cell. The observable motions in the cell were used to estimate the total earthworm movement and the resulting tunnel length. Earthworm motion was detected using particle image velocimetry (PIV) implemented in ImageJ[2]. Results suggest that the earthworm burrowed 20 cm of tunnel in the first three days of the experiment. The earthworm re-used the tunnels for the remainder of the experiment (Supplementary Video S1). An attempt of AE sonification (i.e. transformation of the inaudible AE events in audible data) was conducted using the *playitbyr* R package[3] (Supplementary Video S2). The delay between the acoustics and the image in Supplementary Video S2 is due to the earthworm moving out of view. The AE rate monitored during the whole experiment showed a good correlation to the new tunnel creation (Supplementary Fig. S1) and a poor correlation between earthworm motion and AE rate (Supplementary Fig. S1b). The AE rate was highly correlated to the new tunnels burrowing during the first part of the experiment (days 1-4), and to earthworm displacement during the last part of the experiment (days 5-7) (Supplementary Table S1). The earthworm's absolute rate of motion was initially impeded by the soil, thus the earthworm moved at lower rates when it was forced to burrow. The mechanical burrowing activity produces acoustic emissions; therefore, more AE were measured when the earthworm was moving slower. Towards the end of the experiment, the earthworm begins to move more rapidly after the tunnel system is established. This results in higher rates of absolute motion and reduced rates of AE generation.

Soil structure dynamics and plant root growth in glass cell

Maize root growth was monitored using time-lapse imaging and ImageJ[2] was used for image analysis (image segmentation to identify roots). Even though the glass cell was tilted to exploit root gravitropism towards the front glass, roots also grew on the back. Imaging gave a general idea of the root growth

dynamics, but it can exist a delay between the recorded AE and the observed root growth. It is also obvious that part of the root system was not detected through this imaging technique.

After that the maize seeds were added in the glass cell, the roots grew until the 16th day of the experiment (mean daily root growth rate of 9 cm.day^{-1}; Supplementary Table S2). Growth subsequently slowed down (mean daily root growth rate of 0.3 cm.day^{-1}). Direct imaging estimated a total of 126 cm of roots along the front of the glass cell. Roots where mostly located in the top 15 cm of the glass cell, around the S2 sensor. This could explain why sensor S2 recorded more events than sensor S3, which was further from the root growth activity.

The AE rates remained low during the first six days of the experiment, i.e. two days before the seeds addition and 4 days after (mean AE rate of 267 and 172 EA.day^{-1} for sensors S2 and S3; Supplementary Table S2). The AE rates increased drastically over days 6-15 (mean daily AE rate of 2088 and 452 EA.day^{-1} for sensors S1 and S2). From day 16, the AE rate plateaued until the end of the experiment (mean daily AE rate of 649 and 157 EA.day^{-1} for sensors S2 and S3). Despite agreement between the AE trends and the observable root growth, the correlation between the daily rates of root growth and AE rates were low when considering the whole experiment (R^2 of 0.04 and 0.25 for sensors S2 and S3) (Supplementary Fig. S2). Correlations between AE rates and root growth rate were better when considering the three sub-periods, expected for sensor S2 during days 7-15. This can be due to our limited ability to precisely monitor root growth visually. The root length results from the observable roots in front of the cell. However, more roots were growing into the soil, possibly in contact with the background glass of the cell to which the sensors were connected. This can also explain why we observed a lag between the AE (increasing rate at day six) and the root growth (increasing rate at day two).

To ensure that water movements in the soil (evaporation) did produce negligible AE compared to the root growth, AE were monitored in a glass cell filled with Winzlerboden soil, without seed addition. The conditions were the same as those described in the *Materials and Methods* section for the plants root growth in glass cell. The soil experienced a steady water evaporation, with a maximum cumulative water evaporation of 4 mm after 2.3 days of experiment (Supplementary Fig. S3). The monitored AE had a

mean daily rate of 127 AE.day^{-1}, and was lower than the AE rate measured during the first six days of the experiment with addition of maize roots (Supplementary Table S2). This result support the idea that the water movements in soil did produce AE, but in a lower extend that the root growing process.

AE sonification was also conducted using the *playitbyr* R package[3] (Supplementary Video S3). We detect a lag in Supplementary Video S3 between the observed root growth and the sonified AE due to the roots growing out of view of the camera (in the soil or along the back glass of the glass cell).

Soil structure dynamics and plant root growth in soil columns

During the first two days of the experiment, similar AE rate were registered for upper and lower sensors (S2 and S4 at 5 cm depth and S3 and S5 at 20 cm depth Supplementary Table S3). Mean daily AE rates of 39, 17, 112 and 140 AE.day^{-1} were recorded for the sensors S2, S4, S3 and S5, respectively. From day three to eight, an increase in AE rates was observed in the column with maize (sensors S4 and S5), in which mean daily AE rates of 2720 and 2701 AE.day^{-1} were recorded. In contrast, the AE rates in the control bare soil column stayed stable (sensors S2 and S3, mean daily AE rates of 25 and 142 AE.day^{-1}). During the last two days of the experiment, the daily AE rates in the control column (sensors S2 and S3) maintained similar values to those observed previously (24 and 138 AE.day^{-1}). The daily AE rates in the column with maize decreased but remained higher than the control column and higher than in the beginning of the experiment (264 and 567 AE.day^{-1}).

Results repeatability

The three experiments described in the main text of this paper were re-run in the same conditions as described in the *Materials and Methods* section to ensure the results repeatability. Supplementary Fig. S4 to S7 show the results of the experiment replicates to assess acoustic emissions from a burrowing earthworm in soil, acoustic emissions from plants root growth in a glass cell, and acoustic emissions from growing plants roots using waveguides in soil columns.

Supplementary Fig. S4 shows the results of the experiment replicate to assess acoustic emissions from the burrowing earthworm in soil. The cumulative length of the earthworm's burrow was 15 cm by the end of the experiment (Supplementary Fig S4f), with 13 cm of tunnels created in the first three days of the experiments. The final two cm were progressively burrowed in the final four days. During this replicate experiment, after a first 13 days, the earthworm re-used the existing tunnels but also continued to create new burrows (Supplementary Fig S4e). The earthworm displacement rate started increasing one day after the beginning of the experiment and continued to increase regularly until the end. This leads us to think that the earthworm activity included both tunnel creation and reuse even in the first three days. Acoustic emissions generated by earthworm activity (Supplementary Fig. S4) were dominantly detected by the sensor located at the depth of 20 cm in the cell (sensor S3). This dominance of the lower sensor S3 was due to the burrowing activity local to the lower section of the column (Supplementary Fig. S4b-c). During the first three days of the experiments, the acoustic signature of these events was equally correlated with the cumulative burrowing activity estimated by visual activity and the absolute motion (Supplementary Table S4). This result is consistent with the observed earthworm activity that combine tunnel creation and reuse in this phase. During the last four days of the experiments, the AE events were mostly correlated with the earthworm displacement. These results are consistent with the results shown in the main text, illustrating the consistence of the link between AE signature and earthworm activity.

The acoustic emissions generated by the three plants roots growing in the glass cell were not as pronounced in the replication experiment than in the original experiment (Supplementary Fig. S5). However, the AE events were still detectable and behave characteristically similar to the visually monitored total root length (Fig. S5a-c). Both measurements come to a plateau towards the end of the experiment. The average AE rates measured during the first six days of the experiments (689 and 375 $AE.day^{-1}$ for sensors S2 and S3) where higher than those measured during the twelve last days of the experiment (138 and 71 $AE.day^{-1}$ for sensors S2 and S3) (Supplementary Table S5). This was consistent with the average root growth rate, which was higher during the first six days of the experiments (11 $cm.day^{-1}$) than during the twelve last days of the experiment (2 $cm.day^{-1}$). These results were also

consistent with those presented in the main text. The total root growth slowed down and plateaued around day 11 with an observable total root length of about 92 cm. The total root length estimated in the replicate experiment was lower than in the original experiment, which would explain the lower AE events recorded in the replicate. Moreover, despite the more pronounced appearance of the roots in the replication experiment, comparison of the final root distribution along the front and back faces of the glass cell revealed that there were fewer roots in total for the replicate (and no roots were observed on the back face), resulting in significantly reduced AE in the replicate experiment (Supplementary Fig. S6). The absolute magnitude of the acoustic events detected by the root growth were less than double that of the background noise (thus the normalized events were slightly less than the background noise). The background noise sensor S1 recorded the AE events occurring in the surrounding. It was expose to any occurring disturbance independent to the experiment and its magnitude does not have any significance regarding plant roots growth.

The experimental replicate of the plants root growing in the soil column (Supplementary Fig. S7) were similar in to the results obtained in the original experiment. The magnitude of the AE signatures were slightly lower by comparison, as well as the AE rates (Supplementary Table S6); however, the acoustic signature was characteristically similar. Nevertheless, only four plants grew during this replicate experiment (compare to the twelve in the experiment exposed in the main text). This could explain the lower AE rates recorded in this experiment. Stem heights reached on average 1.5 cm three days after planting, about 6 cm after five days, about 20 cm after nine days, and about 30 cm at the end of the experiment (after thirteen days). Then, the plants grew up a bit slower than in the original experiment but the stem finally reached longer length. Considering the movement of water in the column (Fig S7b-c), we can see that the large changes in the acoustic signatures occur prior to any rapid changes in the water content. Ultimately, the water movement cannot be the source of the rapid generation of acoustic events in this experiment. These results support the hypothesis that the AE rates can be explained by root growth activity in soil and confirm the potentiality of the method to monitor soil bioturbation processes.

Supplementary References

1. R Core Team. R: A language and environment for statistical computing. R Foundation for Statistical Computing, Vienna, Austria. https://www.R-project.org/ (2015)
2. Rasband WS. ImageJ, U. S. National Institutes of Health, Bethesda, Maryland, USA. https://imagej.nih.gov/ij/ (1997-2016).
3. Brown E. playitbyr: Representing and exploring data through sound. R Package: Version 0.2-1, http://playitbyr.org/ (2012)

Supplementary videos legends

Supplementary Video S1. Earthworm displacement estimation in the glass cell (burrowing and use of the tunnels). Dashed circles: sensor S2 and S3 location. Black arrow: entry point of the earthworm in the glass cell. Black points: locations where earthworm movements were detected by time-lapse imaging. Lines: estimation of earthworm trajectories. Time format: Day Hour:Minute:Second.

Supplementary Video S2. Observed earthworms motion in the glass cell with sonification of AE data from sensor S2. Time format: Hour:Minute:Second.

Supplementary Video S3. Plant root growth in the glass cell with sonification of AE data from sensor S2. Time format: Hour:Minute:Second.

Supplementary Figure S1. Relation between daily AE rate and earthworm activities. a) Rate of new tunnels burrowing. Squares: sensor S2 (solid line: $y = 0.9 \times 10^{-3}x - 1.7$). Circles: sensor S3 (dashed line: $y = 5.4 \times 10^{-2}x - 2.2$). b) Daily rate of earthworm displacement. Squares: sensor S2 (solid line: $y = -2.5 \times 10^{-2}x + 364$). Circles: sensor S3 (dashed line: $y = -1.1 \times 10^{-2}x + 339$). White symbols: data for days 1-4; Black symbols: data for days 5-7. The results for sensors S2 and S3 are given after background noise filtering from sensor S1.

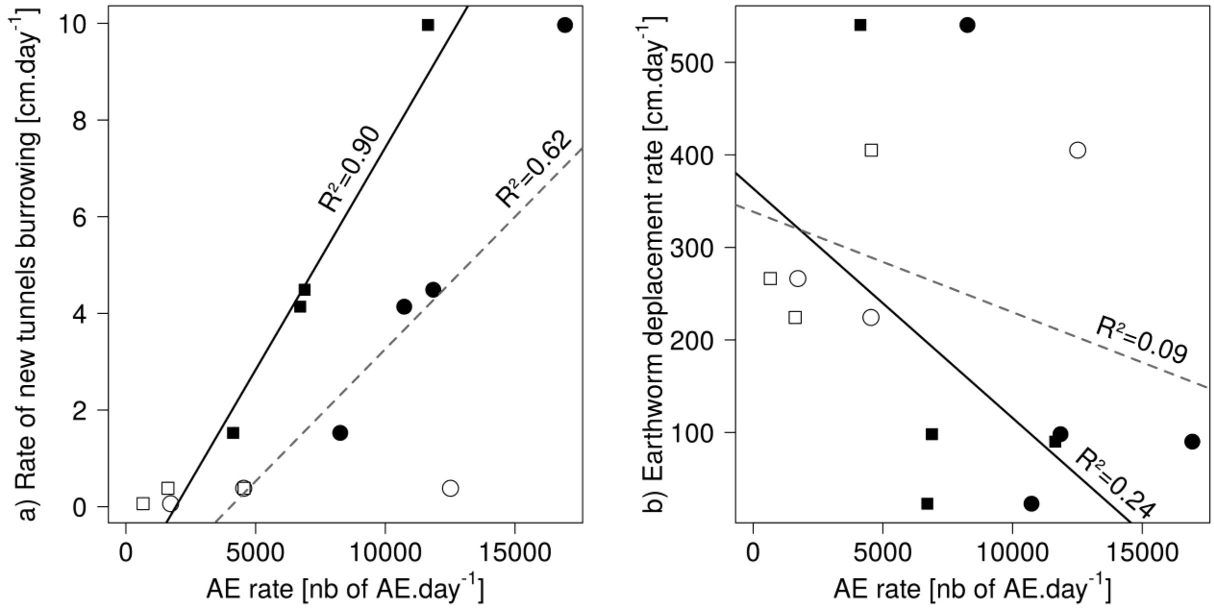

Supplementary Figure S2. Relation between daily AE rate and root growth rate. Solid line for Sensor S2 ($y = 1 \times 10^{-3}x + 5.2$). Dashed line for sensor S3 ($y = 1 \times 10^{-2}x + 2.6$). The results for sensors S2 and S3 are given after background noise filtering from sensor S1

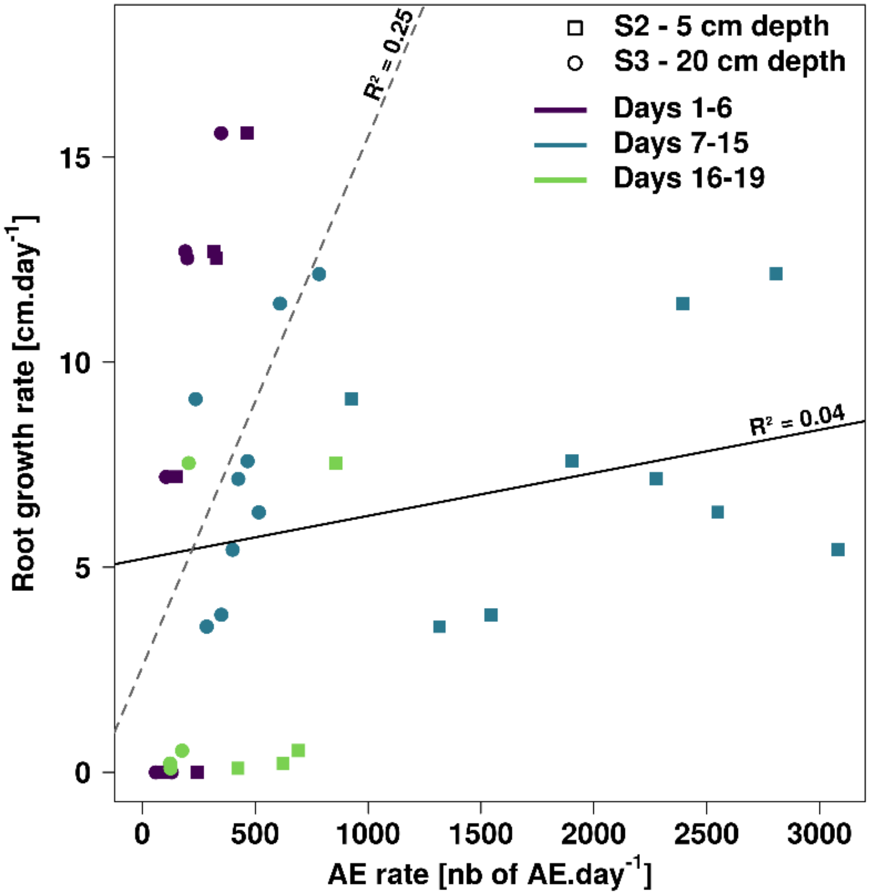

Supplementary Figure S3. AE monitoring during soil evaporation in the glass cell. a) Cumulative number of acoustic events over time, and b) cumulative water uptake over time. The results for sensor S2 are given after background noise filtering from sensor S1.

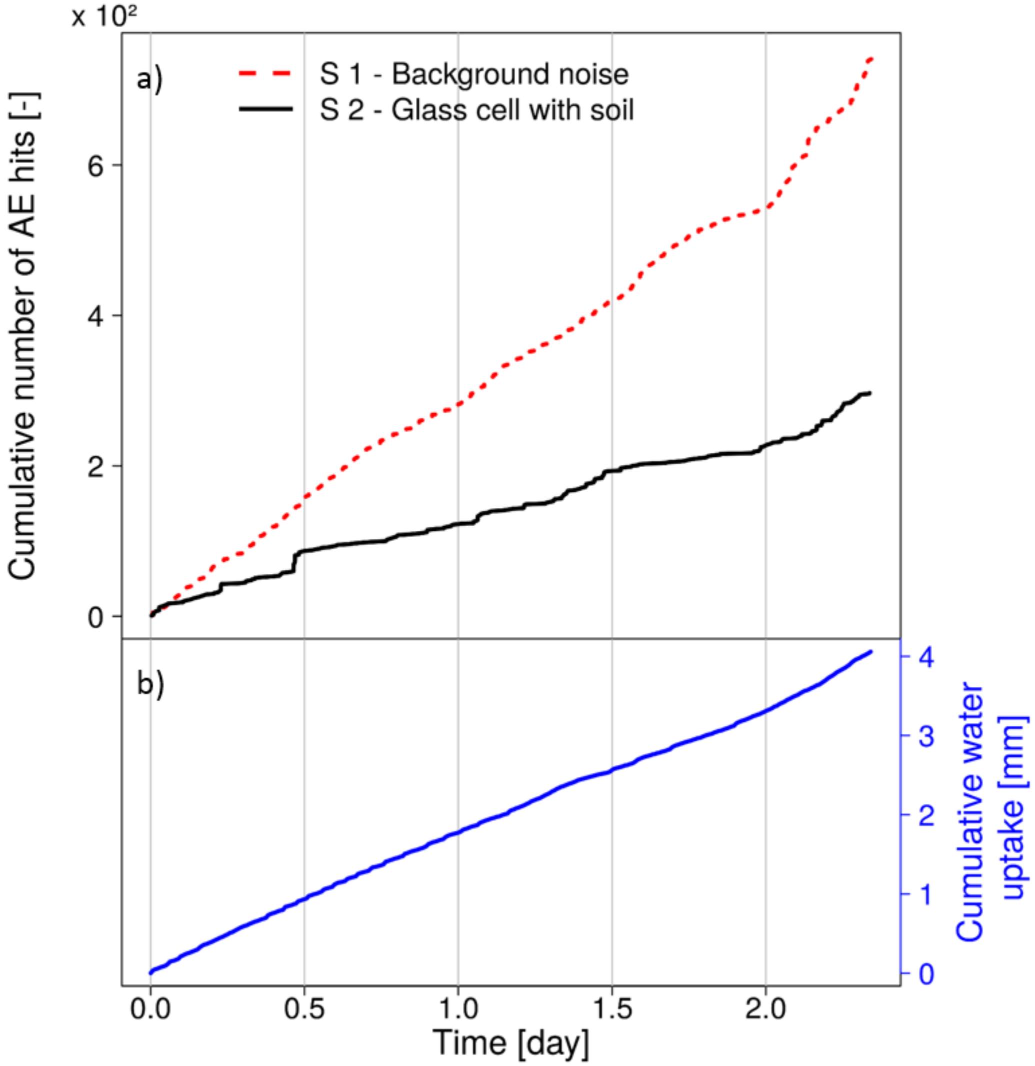

Supplementary Figure S4. AE monitoring and earthworm activity in a soil filled glass cell (replicate experiment). Time-lapse images were taken from the front face of the glass cell for the full duration of the experiment (a: beginning and–b: end of the experiment), where X's indicate the locations of the acoustic sensors. The initial packing (a) was augmented by movement of the earthworm (trajectories illustrated in c) culminating in a final perturbed soil state b). (d) Cumulative acoustic events were monitored during the seven days experiment. The results for sensors S2 to S4 are given after background noise filtering from sensor S1. Total cumulative earthworm motion (e) and total length of new tunnels (f) were determined based on the activity monitored using time-lapse images taken from the front face of the glass cell for the full duration of the experiment.

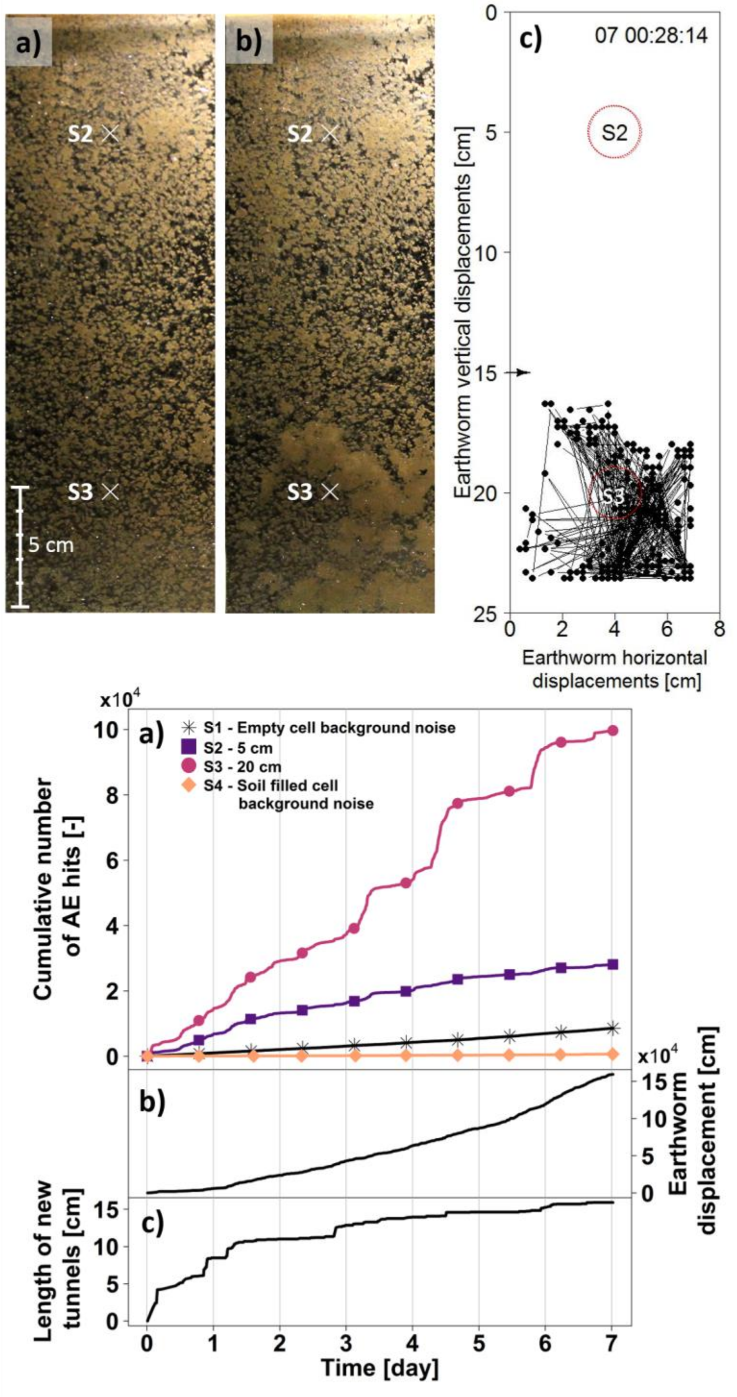

Supplementary Figure S5. AE monitoring during maize roots growing in a soil filled glass cell (replicate experiment). Time-lapse images were taken to monitor maize roots growing in the glass cell from the day the geminated seeds are planted (a) to the last day of the experiment (b), where X’s indicate the locations of the acoustic sensors. Cumulative number of acoustic events were monitored for the three separate acoustic sensors (c). The results for sensors S2 and S3 are given after background noise filtering from sensor S1 (the difference between the raw data and the background noise). Simultaneously, the cumulative water uptake was also monitored (d) as well as the estimated total root length (e) determined with time-lapse images. The vertical dashed line (c-e) denote the time when germinated seeds were planted in the glass cells (first day of the experiment).

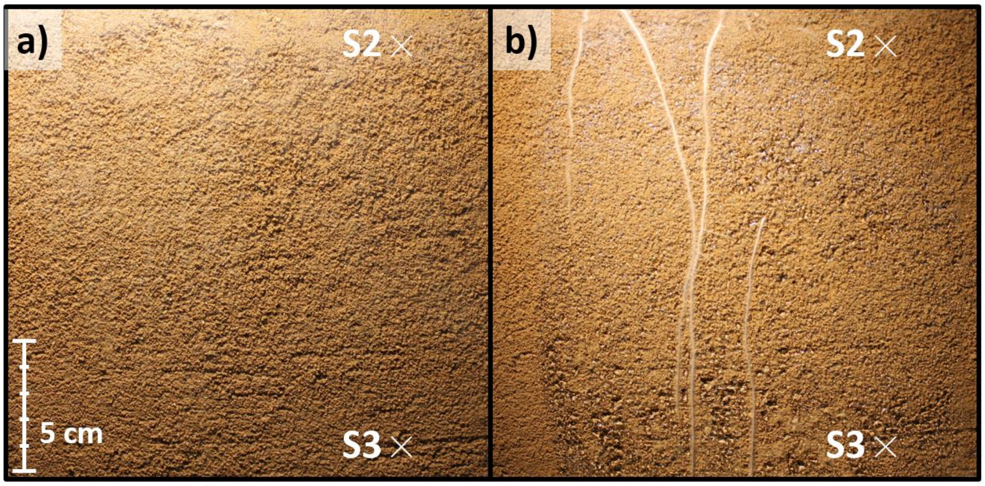

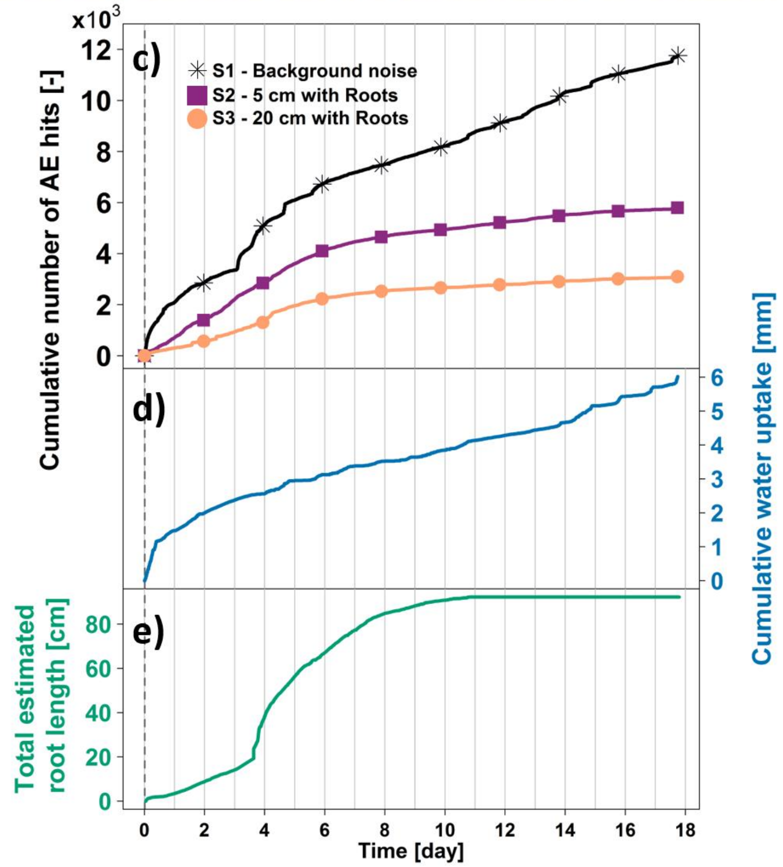

Supplementary Figure S6. Observed maize roots in the soil filled glass cell at the end of the experiments. Images of the front and back faces of the glass cell were taken for the original experiment after 19 days of plant roots growing (a and b) and for the replicate experiment after 19 days of plant roots growing (c and d).

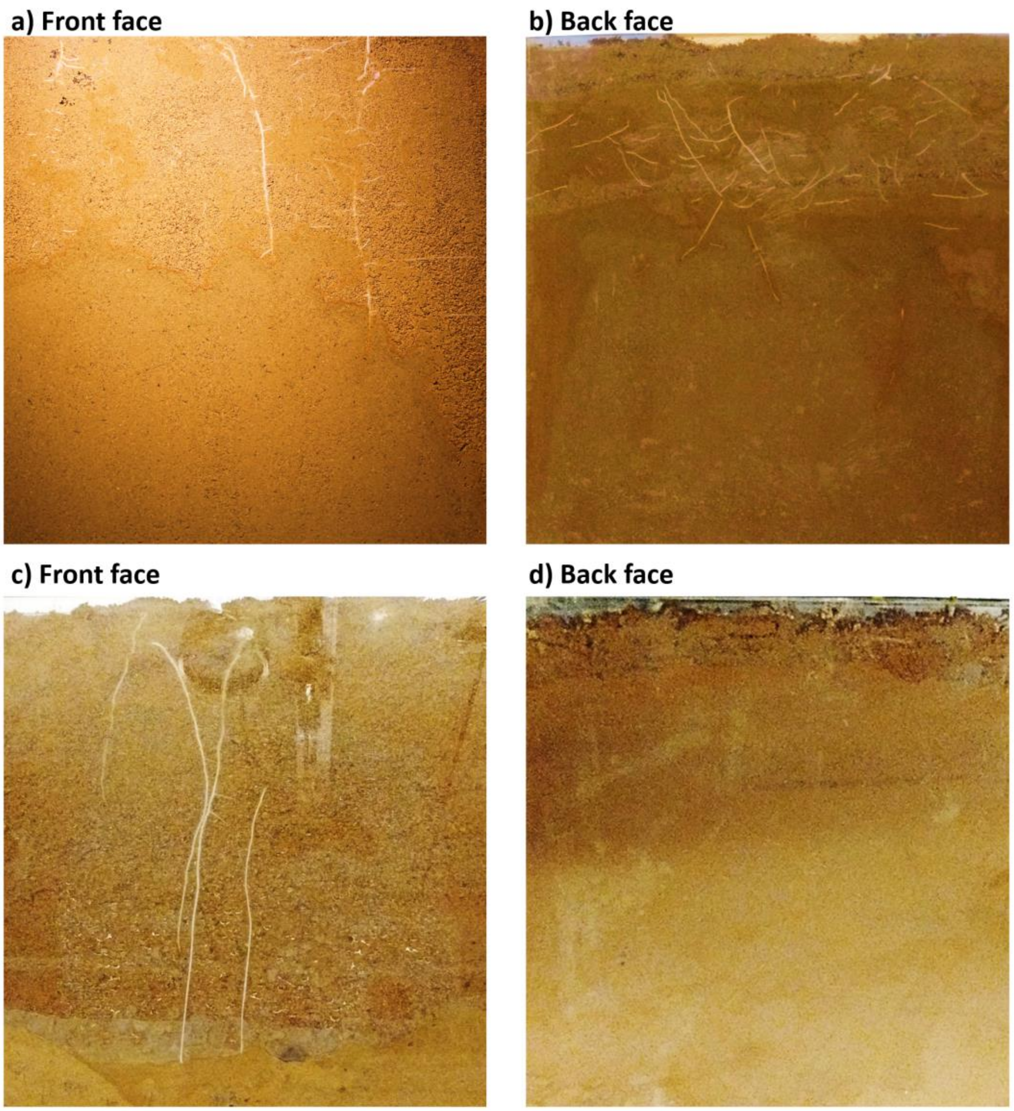

Supplementary Figure S7. Monitoring AE generated by maize roots growing in a soil column (replicate experiment). (a) The results for sensors S2 and S3 are given after background noise filtering from sensor S1(the difference between the raw data and the background noise). Volumetric water content (θv) in the column was measured (b), and the cumulative absolute change in θv over time was computed (hourly time-lapse) (c), representing the absolute change in water content in the column, independently of drying or wetting processes. The vertical dashed line (a-c) denote the time when germinated seeds were planted in the glass cells (at the beginning of the experiment).

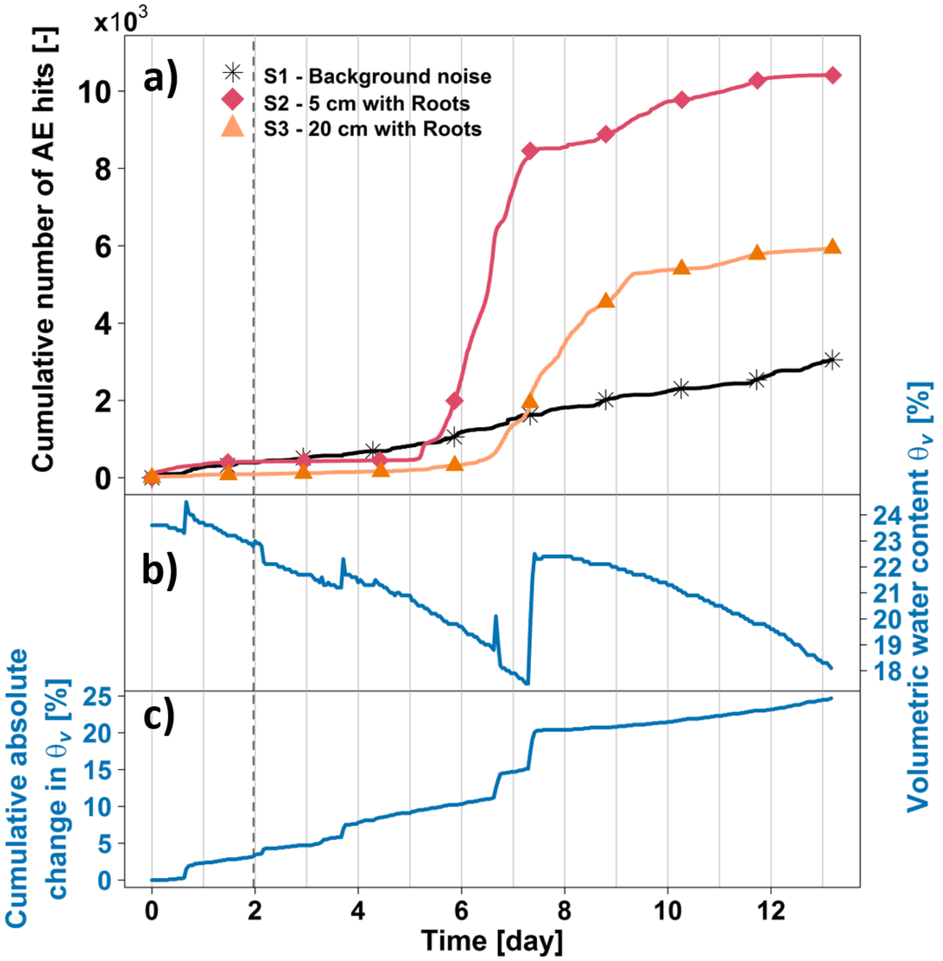

Supplementary Table S1. Comparing statistics of daily AE rates generated by earthworm activity during the first and final half of the experiment. R² give the correlations of AE daily rate with new tunnels burrowing daily rate and earthworm displacement daily rate. The results for sensors S2 to S4 are given after background noise filtering from sensor S1.

		Min.	1st quartile	Median	Mean	3rd Quartile	Max.	SD	nb of AE	R^2 - New tunnels	R^2 - Earthworm displacement
AE rate [AE.day^{-1}]											
Days 1-4	S1	1012	1021	1044	1062	1086	1149	62	4250	-	-
	S2	4134	6070	6802	7346	8078	11640	3130	29382	1.00	0.40
	S3	8261	10110	11290	11940	13120	16930	3646	47766	0.99	0.37
	S4	32	58	69	68	79	102	29	272	-	-
Days 5-7	S1	1215	1350	1486	1429	1536	1586	192	4287	-	-
	S2	661	1140	1620	2279	3088	4555	2029	6836	0.48	0.80
	S3	1723	3132	4541	6259	8526	12510	5596	18776	0.49	0.79
	S4	71	90	109	131	161	213	74	393	-	-
New tunnels burrowing rate [cm.day^{-1}]											
Days 1-4		1.5	3.5	4.3	5.0	5.9	10.0	3.5			
Days 5-7		0.1	0.2	0.4	0.3	0.4	0.4	0.2			
Earthworm displacement rate [cm.day^{-1}]											
Days 1-4		23.0	73.3	94.1	187.9	208.7	540.4	237.4			
Days 5-7		224.3	245.3	266.3	298.6	335.7	405.1	94.6			

Supplementary Table S2. Statistics of daily AE rates and root growth rates measured during the glass cell experiment. R^2 give the correlations between AE daily rate and root growth rate. The results for sensors S2 and S3 are given after background noise filtering from sensor S1.

		Min.	1st quartile	Median	Mean	3rd Quartile	Max.	SD	nb of AE	R^2
AE rate [AE.day^{-1}]										
Days 1-6	S1	234	252	305	338	385	539	117	2029	-
	S2	102	173	280	267	326	463	131	1603	0.66
	S3	60	111	160	172	197	349	101	1033	0.69
Days 7-15	S1	232	341	431	402	472	537	108	3619	-
	S2	927	1544	2275	2088	2549	3080	717	18796	0.08
	S3	236	350	425	452	516	783	169	4071	0.50
Days 16-19	S1	203	213	279	293	358	410	100	1170	-
	S2	423	573	658	649	733	857	180	2595	0.64
	S3	123	125	151	157	183	205	40	629	0.67
Root growth rate [cm.day^{-1}]										
Days 1-6		0.00	1.80	9.87	8.00	12.66	15.59	6.77	-	-
Days 7-15		3.55	5.43	7.16	7.40	9.10	12.15	3.05	-	-
Days 16-19		0.10	0.19	0.37	2.10	2.28	7.54	3.63	-	-

Supplementary Table S3. Statistics of AE measured in the soil columns experiment. The results for sensors S2 to S5 are given after background noise filtering from sensor S1.

		Min.	1st quartile	Median	Mean	3rd Quartile	Max.	SD	nb of AE
AE rate [AE.day^{-1}]									
Days 1-2	S1	150	-	-	161	-	172		322
	S2	33	-	-	39	-	44	8	77
	S3	81	-	-	112	-	143	44	224
	S4	15	-	-	17	-	19	3	34
	S5	129	-	-	140	-	150	15	279
Days 3-8	S1	89	132	217	199	264	291	85	1196
	S2	13	19	25	25	32	39	10	152
	S3	117	120	122	142	161	200	36	854
	S4	735	1406	2225	2720	2807	6999	2251	16318
	S5	284	965	2828	2701	4257	5190	2043	16207
Days 9-10	S1	107	-	-	140	-	173	47	280
	S2	21	-	-	24	-	27	4	48
	S3	116	-	-	138	-	160	31	276
	S4	129	-	-	264	-	399	191	528
	S5	243	-	-	567	-	890	457	1133

Supplementary Table S4. Comparing statistics of daily AE rates generated by earthworm activity during the first and final half of the replicate experiment. R^2 give the correlations of AE daily rate with new tunnels burrowing daily rate and earthworm displacement daily rate. The results for sensors S2 to S4 are given after background noise filtering from sensor S1.

		Min.	1st quartile	Median	Mean	3rd Quartile	Max.	SD	nb of AE	R^2 - New tunnels	R^2 - Earthworm displacement
AE rate [AE.day^{-1}]											
Days 1-3	S1	1012	1021	1045	1063	1086	1149	62	4250	-	-
	S2	3130	3693	5140	5046	6493	6773	1811	20182	0.43	0.42
	S3	8173	12917	14556	13514	15153	16769	3710	54054	0.25	0.32
	S4	32	58	69	68	79	102	29	272	-	-
Days 4-7	S1	1215	1351	1486	1429	1536	1586	192	4287	-	-
	S2	1542	1850	2158	2633	3178	4198	1390	7898	0.25	0.93
	S3	5195	10390	15584	15196	20197	24809	9813	45588	0.01	0.68
	S4	71	90	109	131	161	213	74	393	-	-
New tunnels burrowing rate [cm.day^{-1}]											
Days 1-3		1.8	2.2	2.5	4.3	5.5	8.5	3.6			
Days 4-7		0.6	0.7	0.7	0.8	0.8	1.1	0.2			
Earthworm displacement rate [cm.day^{-1}]											
Days 1-3		58.5	118.4	178.2	142.9	185.1	191.9	73.4			
Days 4-7		206.3	226.4	278.9	290.6	343.1	398.4	87.9			

Supplementary Table S5. Statistics of daily AE rates and root growth rates measured during the glass cell replicate experiment. R^2 give the correlations between AE daily rate and root growth rate. The results for sensors S2 and S3 are given after background noise filtering from sensor S1.

		Min.	1st quartile	Median	Mean	3rd Quartile	Max.	SD	nb of AE	R^2
AE rate [AE.day^{-1}]										
Days 1-6	S1	481	706	870	1128	1574	2088	650	6768	-
	S2	528	683	697	689	719	805	90	4131	0.03
	S3	265	297	333	375	384	634	135	2250	0.76
Days 7-18	S1	278	345	395	416	478	611	103	4991	-
	S2	48	87	137	138	159	332	77	1661	0.84
	S3	22	53	64	71	71	169	38	849	0.83
Root growth rate [cm.day^{-1}]										
Days 1-6		3.37	5.31	8.04	11.16	16.23	24.16	8.31	-	-
Days 7-18		0.00	0.00	0.00	2.11	2.78	10.31	3.44	-	-

Supplementary Table S6. Statistics of AE measured in the soil columns replicate experiment. The results for sensors S2 to S3 are given after background noise filtering from sensor S1.

		Min.	1st quartile	Median	Mean	3rd Quartile	Max.	SD	nb of AE
AE rate [AE.day^{-1}]									
Days 1-2	S1	114	155	196	196	236	277	115	391
	S2	59	133	207	207	280	354	209	413
	S3	16	30	44	44	58	72	40	88
Days 3-5	S1	130	132	134	144	152	169	21	433
	S2	10	12	14	15	18	21	6	45
	S3	27	33	39	36	41	43	8	109
Days 6-10	S1	174	253	293	283	342	355	73	1417
	S2	432	748	1096	1855	2121	4876	1804	9273
	S3	131	616	1033	1035	1297	2099	741	5176
Days 10-14	S1	59	129	213	202	286	325	120	809
	S2	2	49	153	170	274	374	169	681
	S3	23	74	120	141	186	300	118	562
